# Molecular composition of soil organic matter (SOM) regulate qualities of tobacco leaves

**DOI:** 10.1038/s41598-022-19428-6

**Published:** 2022-09-12

**Authors:** Xu Zhai, Long Zhang, Ruofan Wu, Mei Wang, Yanxiang Liu, Jiapan Lian, Mehr Ahmed Mujtaba Munir, Dan Chen, Lei Liu, Xiaoe Yang

**Affiliations:** 1 Key Laboratory of Environment Remediation and Ecological Health, Ministry of Education, College of Environmental Resource Sciences, Zhejiang University, Hangzhou, 310058 China; 2 Bijie Branch Company of Guizhou Tobacco Company, Guizhou, 551713 China

**Keywords:** Biochemistry, Carbohydrates, Lipids

## Abstract

Soil organic matter (SOM) is of vital importance to soil health, and also plays a crucial role in the quality of the crops such as tobacco. However, the link between tobacco quality and SOM chemical compositions is still not well understood. To fill the information gap, we analyzed the quality of tobacco leaves and the corresponding SOM molecular compositions by electrospray ionization (ESI) coupled with Fourier transform ion cyclotron resonance mass spectrometry (FTICR-MS), that were collected from six different sites in Bijie, Guizhou Province, China. The tobacco quality variedin six sites based on their chemical compositions. SOM compounds had a remarked impact on the quality of tobacco leaves and a distinct difference in SOM composition between low-quality and high-quality tobacco leaves was observed as well. Specifically, 105 common molecular formulas were detected in three SOM compounds of high-quality tobacco, which were more than those in low-quality samples. Although amino sugar, proteins, lipids, tannins, and carbohydrates had a collective influence on the chemical composition of tobacco leaves, the effect contributed by amino sugar and tannins was more prominent. In summary, fully understanding the association between tobacco chemical composition and SOM compounds can provide new insight into the regulation of tobacco quality and the sustainable development of agriculture.

## Introduction

SOM is defined as “the sum of all naturally derived organic materials present” and is quite complex, with thousands of combinations^1^. SOM contributes significantly to soil development, microbial metabolism, and cycling and distribution of nutrients^2^. Research on characteristics of SOM components have started since the early nineteenth century and recently, scientists found that the critical components of SOM include pyrogenic C (the so-called black carbon; BC), humic substances, and products of human activities^3^. The concentration of SOM is highly susceptible to anthropogenic activities, such as cultivation, fire and acidic precipitation, and climate change, while these changes of SOM may influence the growth and development of crops in turn^2^. SOM become one of the most complex mixtures of organic compounds since their formation and accumulation is a long-term evolution process under specific climatic and biological environmental conditions^4^. Moreover, SOM is a key indicator of soil quality and fertility that can impact the quality and quantity of crops^5^.

To explore SOM chemical composition, many advanced detection technologies have been proposed including nuclear magnetic resonance (NMR) technology, Fourier Transform Infrared (FTIR) Spectroscopy,fluorescence metrics, Excitation Emission Matrix (EEM) fluorescence, and so on^6–9^. Noteworthily, Fourier Transform Ion Cyclotron Resonance Mass Spectrometry (FTICR-MS) has become a powerful tool in analyzing the complex organic mixtures with ultra-high resolution. For example, in 1997, Anne Fievre first used FTICR-MS technology to study humic and fulvic acids components^10^. Since then, FTICR-MS technology has been widely used to study the soil organic matter composition at the molecular level. Likewise, FTICR-MS provided the molecular-level insights into the degradation of SOM in the Arctic under global warming^11^, which would predict carbon fluxes and cycling in Arctic soils better. In addition, the application of FTICR-MS successfully identified significant changes of SOM chemical compounds with constant SOM content after planting a cover crop between periods of regular crop production^12^. Similarly, the dramatic impact of fire on the SOM composition can be more detailedly determined by than other techniques^13^. Therefore, applying FTICR-MS technique to characterize SOM components in agricultural soil will advance our understanding of the possible link between soil SOM composition and crop productivity and quality.

Tobacco is an important cash crop in China with a tax profit of 1280.3 billion yuan and a total financial value of 1203.7 billion yuan. Tobacco leaf quality is vulnerable to plant species, environmental factors and soil conditions. It is difficult to find out the low-nicotine species and more than 10 years of breeding and testing would be required to apply the new species. The main factor affecting tobacco quality is soil properties that supplies most of the nutrients plant need for growing. It has been reported that soil properties, including SOM content, soil nitrogen concentration, soil pH and soil microbes have a synergistic effect on tobacco quality. For instance, the diversity of the bacterial community increased when the concentration of organic matter rose^14^ and the increase of microbial diversity and the alter of their distribution in soil can improve tobacco quality^15^. Hu also found that the rich content of soil organic carbon had a positive effect on the quality of tobacco^16^. The tobacco soil containing 3% organic matter was considered to be high-quality soil and also had high catalase activity^14^.The nitrogen content in plant-growing soils should be in an appropriate range. High concentrations of nitrogen can induce excessive growth of tobacco, leading to low accumulation of secondary metabolites (including nicotine, phenols, terpenes, alcohols and lipids) in tobacco^17^. The sugar to nicotine ratio and nitrogen to nicotine ratio may be uncoordinated under the condition of high content of nitrogen in soils^14^. SOM compounds are largely composed of nitrogen compounds, which may impact tobacco quality directly. However, the current study related to tobacco quality just classified the nitrogen compounds into labile soil N, mid-active soil N and inert soil N^18^. Up to now, there is still no insight into the relationship between tobacco quality and the corresponding SOM compounds in soil.

In this study, we hypothesized that the SOM fraction might affect the chemical composition of tobacco leaves and decide the quality of tobacco. Hence, we analyzed the chemical composition of tobacco leaves and the matching SOM compounds jointly using ESI coupled with FTICR-MS. The objective of our research was to (i) compare the SOM chemical composition from tobacco-growing soils of different tobacco qualities; (ii) investigate the relationship between the chemical composition of tobacco leaves and SOM compound types. The results of the study could lead to an appropriate strategy to improve tobacco quality.

## Results

### Soil carbon, nitrogen, and SOM contents

**Figure 1:**
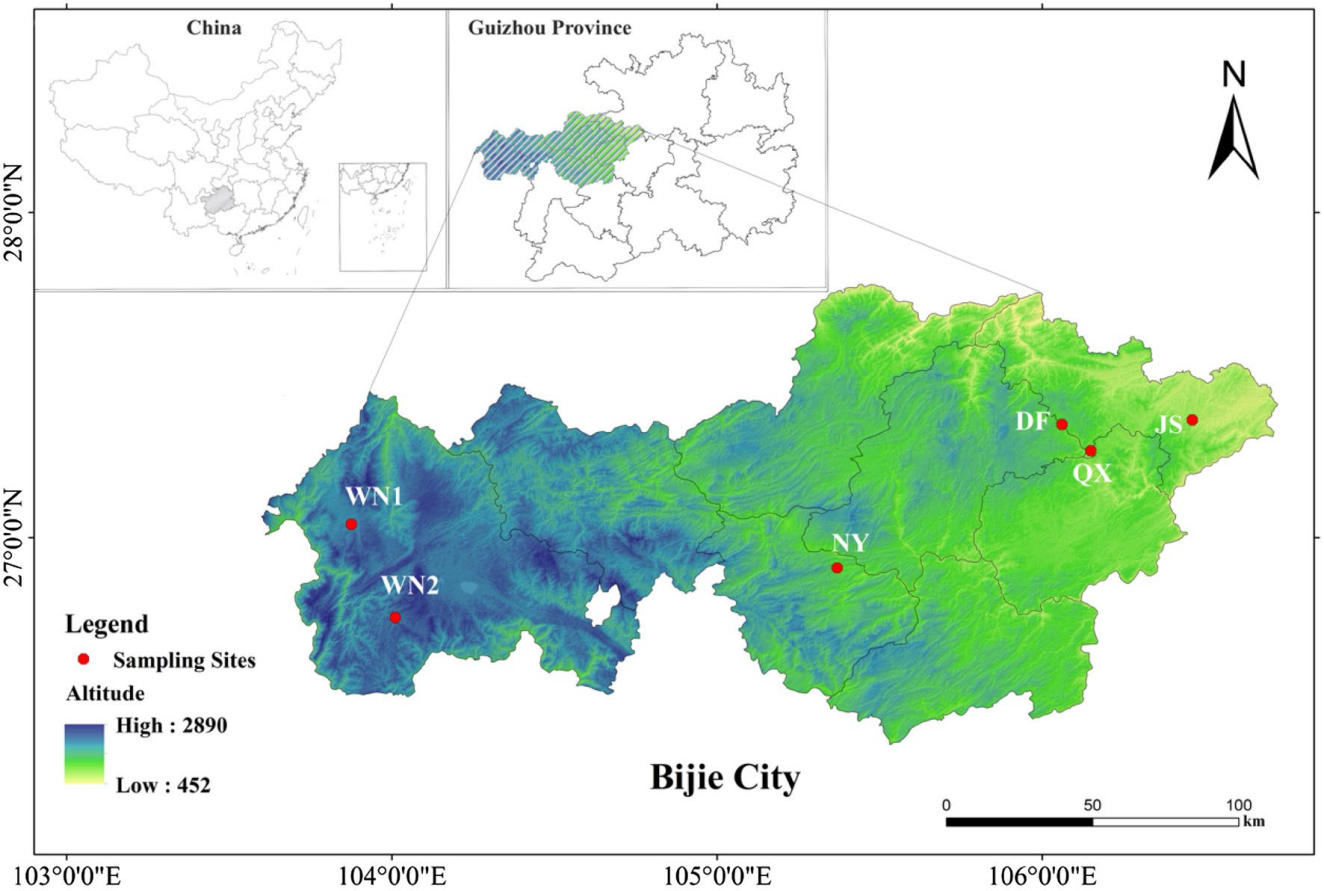
The location of sampling sites in Bijie City, Guizhou Province, China.WN1,2: Wei Ning1,2; NY: Na Yong; DF: Da Fang; QX: Qian Xi; JS: Jin Sha.

The nitrogen, carbon, and SOM content in soils were different in these six sampling sites (Table 1). WN1 and DF had significantly lower nitrogen, carbon, and SOM content than other sites (*P* < 0.05). The nitrogen content in different soils ranged from 0.06 to 0.21% and the carbon content was from 0.59 to 3.86%. The SOM contents in six sites were significantly different. QX had the highest SOM content (55.17 g/kg), whereas WN1 had the least (17.72 g/kg). These two sampling sites were located in the west (WN1) and east (QX) of Bijie City (Fig. 1). Not only did they differ in altitude, but they also shew significant differences in pH values. WN1 and WN2, located in the same county, had similar elevation and pH (Table S1), but they presented significantly different SOM contents.

**Table 1:** The nitrogen, carbon, and SOM contents of soil samples collected in six sites.

Samples	Nitrogen content (%)	Carbon content (%)	C/N Ration	SOM content (g/kg)
WN1	0.06c	0.59d	9.92b	17.72d
NY	0.21a	3.86a	18.50a	36.25c
DF	0.13b	1.12d	8.39c	22.38d
JS	0.16a	1.50d	9.54b	42.43c
QX	0.21a	2.26b	11.15b	55.17a
WN2	0.16a	1.93c	11.73b	48.30b

Different lowercase (a, b, c, d) represent significant differences at *P* < 0.05.

### Tobacco leaves chemical composition.

**Figure 2:**
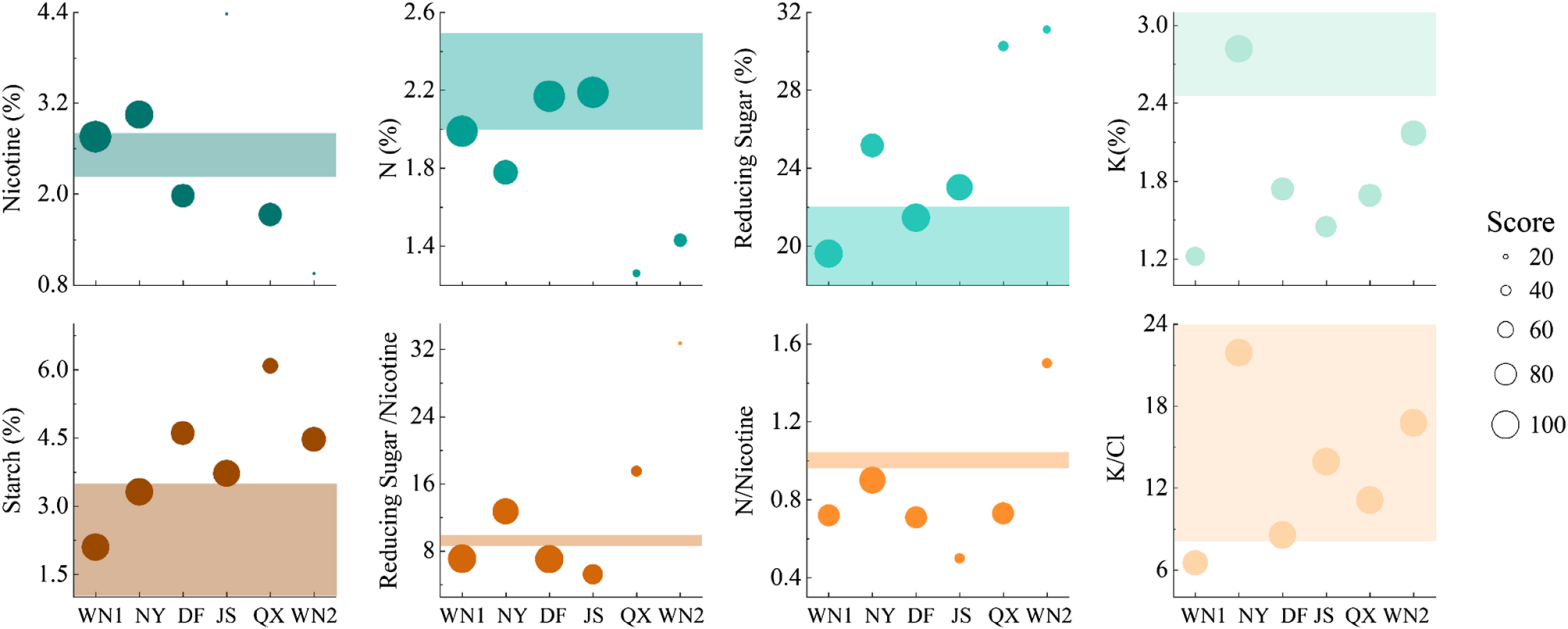
The chemical composition of tobacco leaves and their corresponding score based on the evaluation system. The size of the bubble represents the score of each index. The background filled with color on the graph is the area with a perfect score.

Sample WN1, NY and DF had moderate nicotine contents that near the appropriate range (Fig. 2). The nicotine contents of JS and WN2 were far blow the standard value range. The nitrogen (N) and reducing sugar contents may affect the taste and smell of tobacco as well and Sample WN1, DF, JS had appropriate N and reducing sugar contents. The combustibility of flue-cured may increase with higher potassium (K) content and Sample NY had the highest content. High-quality flue-cured tobacco requires a starch content of less than 5% and only the Sample QX exceeded this range (Fig. 2).

The ratio of reducing sugar to nicotine, nitrogen to nicotine, and potassium to chlorine can reflect the relationship between different chemical compositions and affect the taste and smell of tobacco. WN1, NY, DF had higher points of these ratios than others. However, all the sites didn’t perfectly meet the standard of reducing sugar to nicotine and nitrogen to nicotine. In general, after combining all indicators in the evaluation system of chemical components of flue-cured tobacco, the outcome showed that the Sample WN1, NY, DF could be classified as high-quality tobaccos while Sample JS, QX, and WN2 were considered low-quality ones (Fig. S1).

### The number of peaks extracted by the three solvents

**Figure 3:**
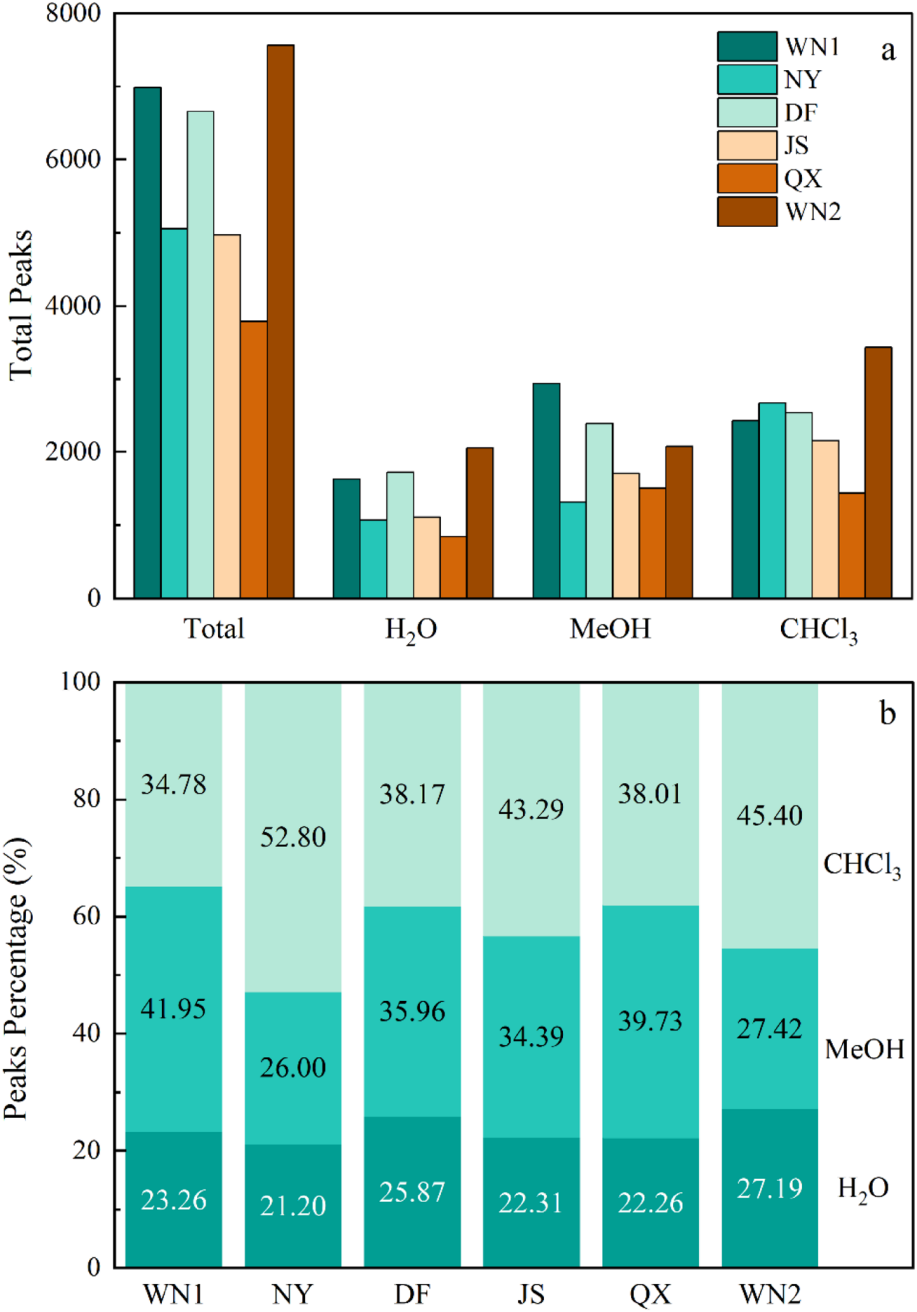
Peak patterns of SOM compounds in soil samples extracted sequentially by H_2_O, MeOH, and CHCl_3_. (**a**) The number of total peaks in soil samples collected in six sites. (**b**) The percentage of peaks extracted by H_2_O, MeOH, and CHCl_3_.

The number of total peaks among different samples ranged from 3783 to 7558 and WN1 and WN2 had the most peaks (Fig. 3). These two samples were collected from Wei Ning County with different tobacco qualities (Fig. 2). Generally, MeOH and CHCl_3_ can extract more compounds than H_2_O in each SOM sample (Fig. 3), accounting for over 70% of total peaks. In the water extract, WN2 had the most peaks (2055) and QX had the lowest ones (842). However, the percentage of peaks extracted by H_2_O ranged from 21.20 to 27.19%, showing similar trends in various samples. For the MeOH extract, WN1 had the most peaks (2930), followed by DF (2391) and WN2 (2072), while NY had the minimum peaks (1313). The compounds extracted by MeOH in WN1 accounted for 41.95% of total peaks. In the CHCl_3_ extracts, only NY accounted for over 45% of total peaks.

### The magnitude-weighted parameters of SOM formulas extracted by different solvents

The magnitude-weighted parameters were calculated based on the formulas and relative intensity (Table S2). First of all, we compared these parameters extracted by different solvents. For the (H/C)w, (N/C)w, (O/C)w, and C#w, it was clear that (H/C)w and C#w in water extracts were lower than those in MeOH and CHCl_3_ while the (N/C)w and (O/C)w had opposite results. Differently, MeOH extracts had a higher degree of unsaturation than the other two extracts, which was decided by DBE. Water extracts had higher (DBE/C)w and DBE/C ratios, representing a high density of C=C double bonds and revealing aromatic or condensed aromatic structures. Also, the Ai-mod can distinguish non-aromatic, aromatic, and condensed aromatic compounds. The Ai-mod in H_2_O extracts was higher than in other extracts. As a result, the percentage of condensed aromatic in water extracts was higher than those in other extracts in all samples. The non-aromatic accounted for the vast majority of total formulas (45.3–86.86%) while aromatic for the least (4.49–15.53%).

### Differences in SOM compounds type

**Figure 4:**
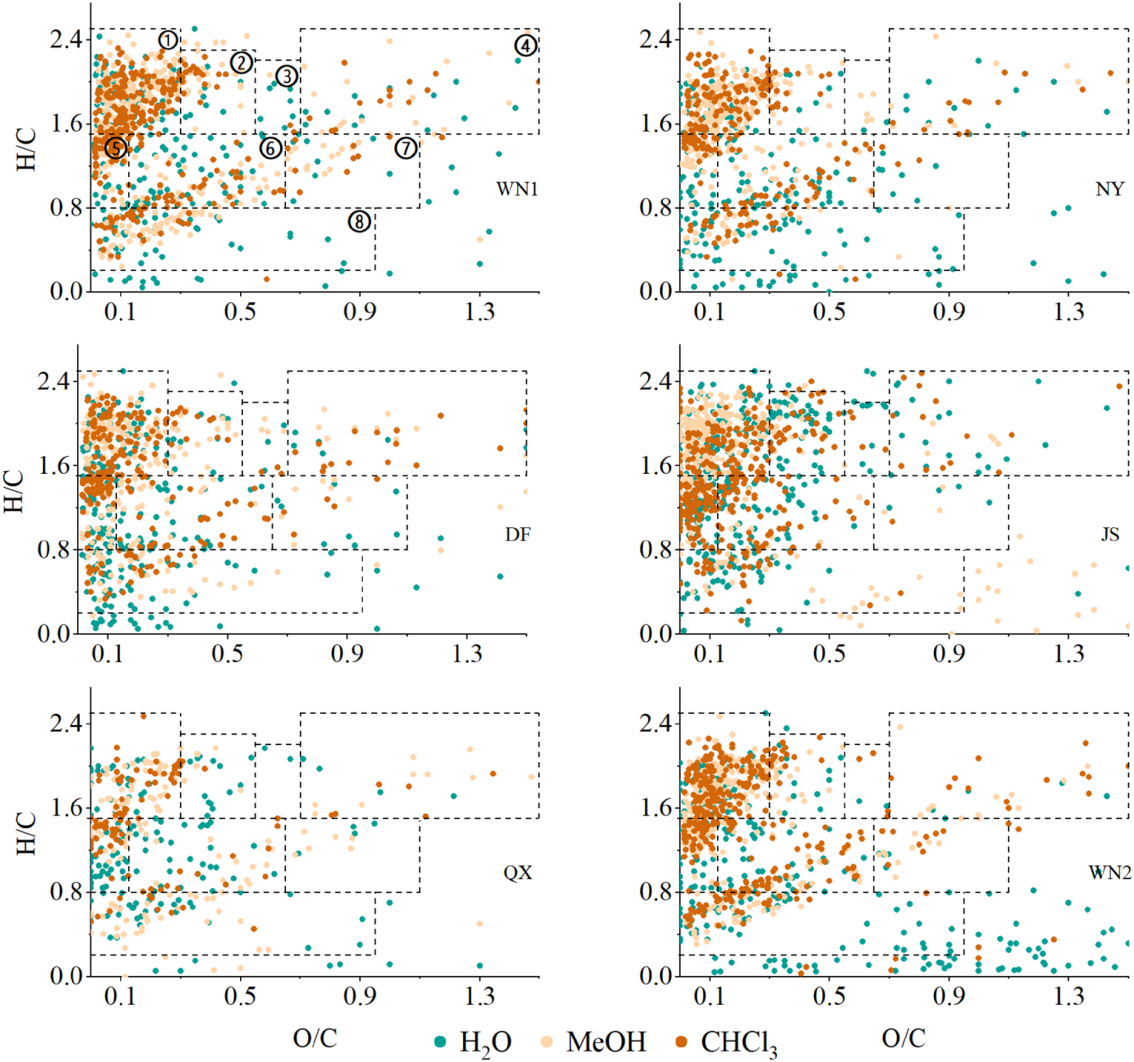
The chemical composition of SOM compounds of different soil samples. Colors indicate different extracts. Different numbers on dotted box in WN1 represent different compounds that are appropriate for all the figures: ① Lipids, ② Proteins, ③ Amino Sugars, ④ Carbohydrates, ⑤ Unsaturated hydrocarbons, ⑥ Lignin, ⑦ Tannins, ⑧ Condensed hydrocarbons.

**Figure 5:**
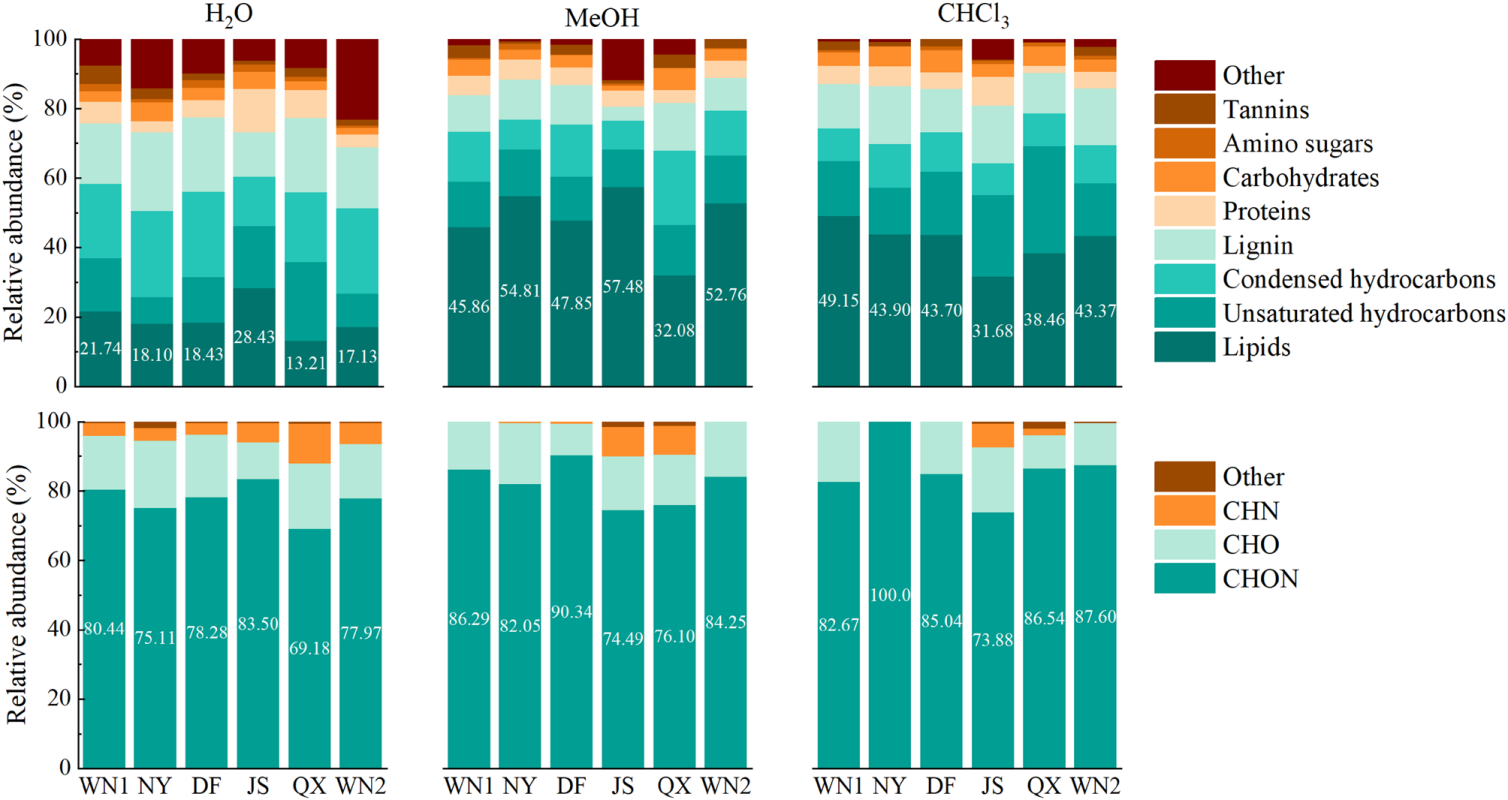
The relative abundances of SOM compounds sequentially extracted by H_2_O, MeOH, and CHCl_3_ determined by FT-ICRMS.

The SOM compounds can be classified into eight different types according to their O/C and H/C ratio, including amino sugars, carbohydrates, condensed hydrocarbons, lignin, lipids, proteins, tannins, and unsaturated hydrocarbons^19^, which is shown in the Van Krevelen diagram (Fig. 4). Generally, all the compounds extracted by three solvents were mainly composed of lipids, unsaturated hydrocarbons, condensed hydrocarbons, and lignin, accounting for an average of 85% in polar extracts and 75% in non-polar extracts of total compounds, respectively (Fig. 5). In the H_2_O extracts, lipids, unsaturated hydrocarbons, and condensed hydrocarbons accounted for about 60% of the total compounds, and each of them had a similar percentage. However, the lipids alone accounted for over 40% in most MeOH and CHCl_3_ extracts (Fig. 5). For the different quality of tobacco, the difference was much smaller among high-quality ones than lower ones.

Also, we grouped the formulas based on their elemental composition, including CHNO, CHN, CHO and others. All the extracts were mainly composed of CHNO, ranging from 69 to 100% (Fig. 5). The compounds extracted by H_2_O were more complex than those of MeOH and CHCl_3_. Among the fractions extracted by water, each sample contained "others" components, and the relative abundance of CHN was higher than that of the other two solvent-extracted fractions. Among them, sample JS contained the highest CHON substance content of 83.5%. In contrast, DF had the highest CHON content (90.34%) in the MeOH-extracted fractions, and WN14 and WN2 did not contain CHN and other substances.The relative abundance of CHON compounds was higher in the NY, QX and WN2 regions obtained in the fractions extracted using CHCl3. Samples WN, NY and DF contained only CHO and CHON fractions, which were also areas of better tobacco quality.

### Common compounds of tobacco planting soils of different qualities

**Figure 6:**
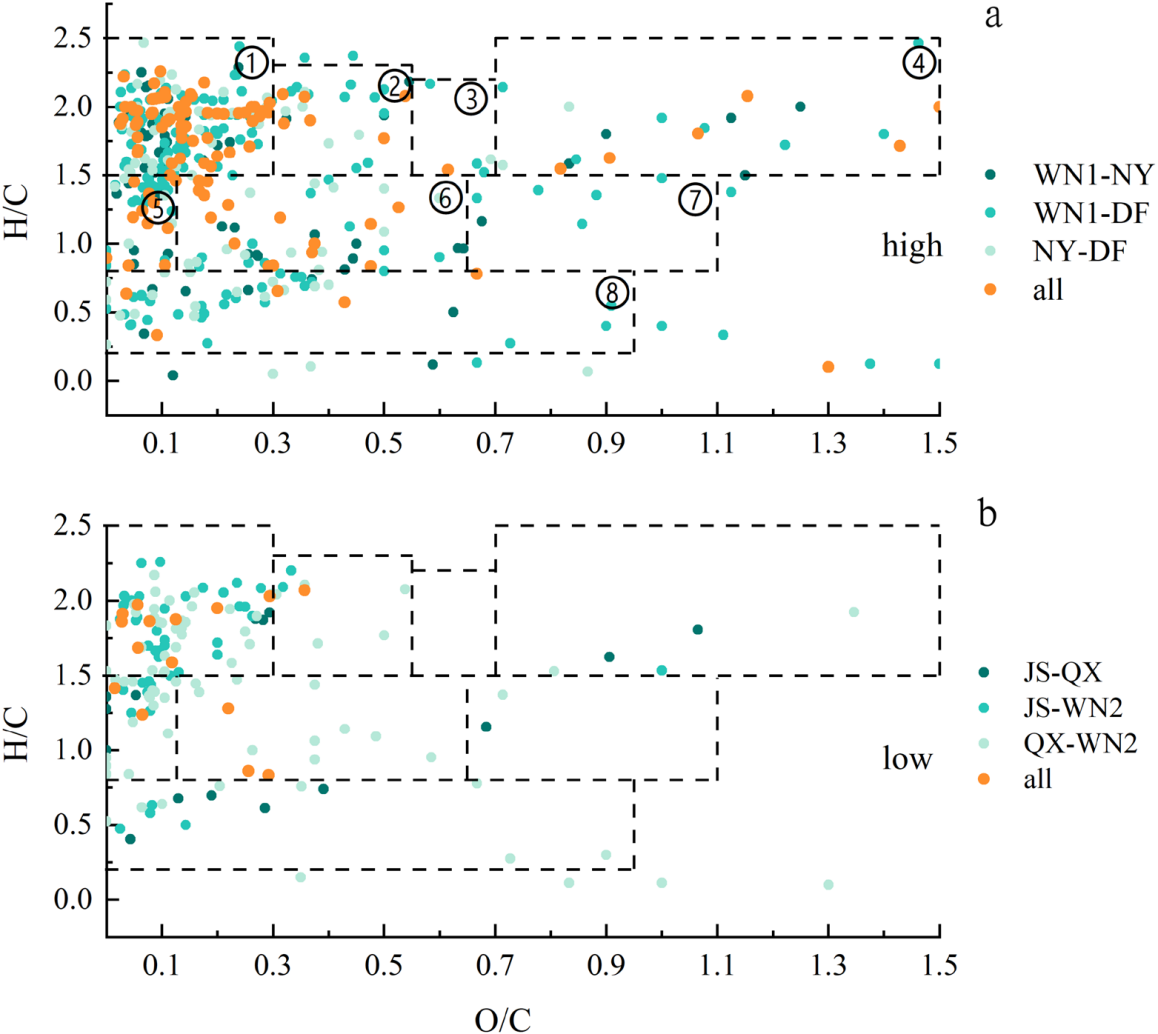
Van Krevelen diagrams of common SOM formulas. (**a**) Common formulas in high-quality soils. (**b**) Common formulas in low-quality soils. Different colors indicate common fractions between two or three samples. Different numbers on dotted box in high represent different compounds that are appropriate for both figures: ① Lipids, ② Proteins, ③ Amino Sugars, ④ Carbohydrates, ⑤ Unsaturated hydrocarbons, ⑥ Lignin, ⑦ Tannins, ⑧ Condensed hydrocarbons.

Van Krevelen plots were used to show common compounds, which means the same formulas found in in high and low quality soils based on O/C and H/C ratios (Fig. 6a,b). In the high quality tobacco soils, 169, 267 and 186 common compounds were found between WN1-NY, WN1-DF and NY-DF, representing 4.77%, 7.53% and 5.25% of the total formulation, respectively (2992). Among three soils of high-quality tobacco, the number of common compounds was 105, accounting for 2.96% of the total formulas (orange dots in Fig. 6a). In the soils of low quality plants, the number of common compounds was much less than in the high quality ones, representing only 0.56% (2549) of the total formulation (Fig. 6b).

Among the three common components of high-quality tobacco soils, except for tannins, all compound types were observed, including lipids, proteins, amino sugars, carbohydrates, unsaturated hydrocarbons, lignin, and condensed hydrocarbons. Among them, lipids account for about 50%, unsaturated hydrocarbons, lignin and condensed hydrocarbons for 10%, and proteins and carbohydrates for 5%. In the low quality soils, only lipids, proteins, Unsaturated hydrocarbons and Lignin were found to be common in all three samples, and lipids constituted the majority (66.67%).

**Figure 7:**
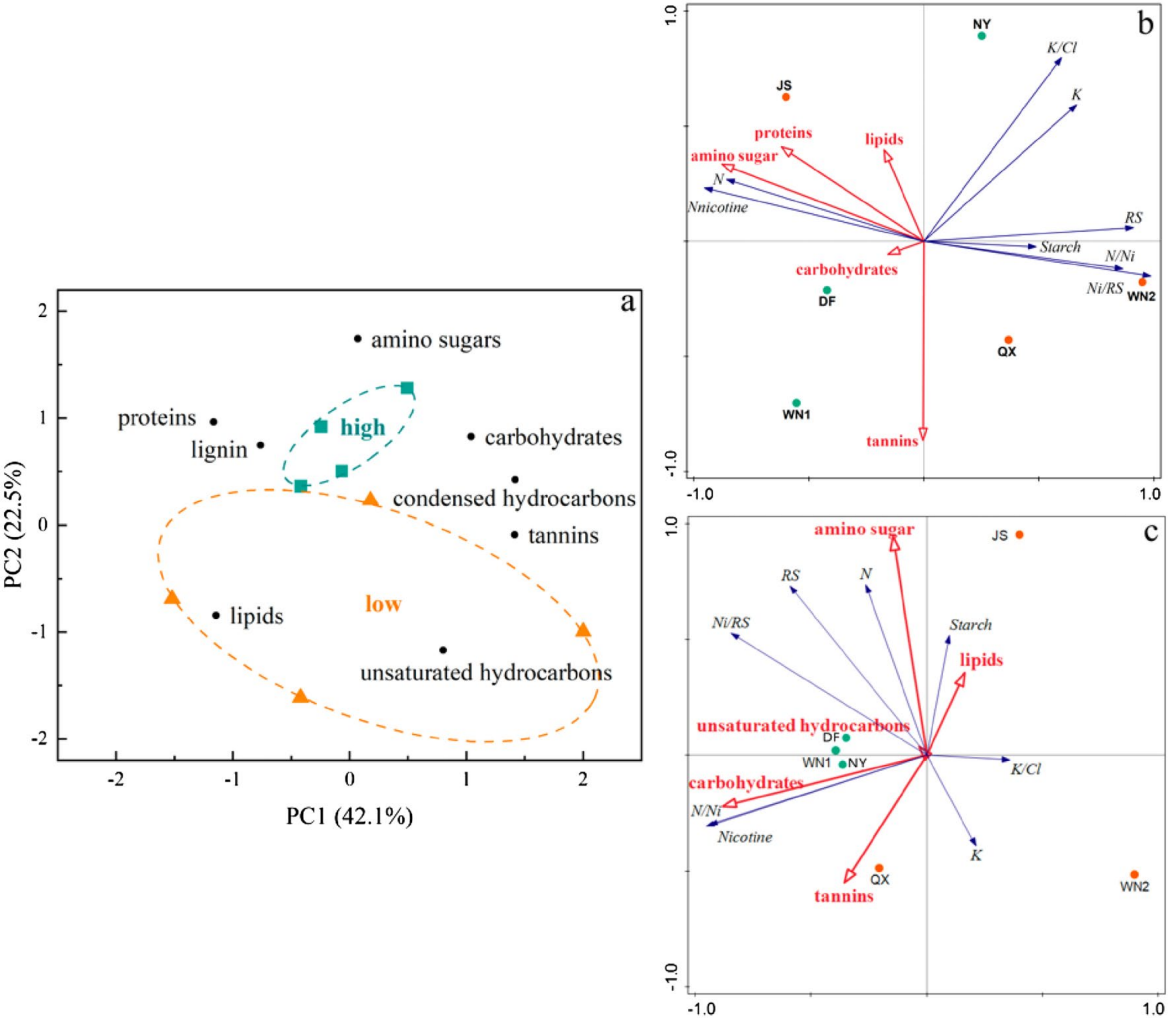
PCA and RDA results based on tobacco chemical composition and SOM compounds. (**a**) PCA of common compounds and their types. (**b**) Ordination biplot based on RDA of tobacco chemical composition and SOM compounds. Tannins and amino sugar were identified as significant factors and included in the model. (**c**) Ordination biplot based on RDA of tobacco chemical composition scores and SOM compounds. Amino sugar were identified as significant factors and included in the model.

Principal component analysis (PCA) was performed to determine the critical parameters affecting the common compounds. Figure 7 a showed green and orange data points were scores for the samples, while the black data points were the loadings from each variable. PC1 explained 42.1% of the total variance and PC2 explained 22.5%. A clear separation was observed between common compounds of different quality plants. The distance between each point determined the degree of similarity and difference of the various compounds between the points. As shown, the score of higher quality compounds was positive on the PC2 axis while the low-quality ones scattered in three quadrants, indicating that good-quality soil compounds were more similar than low-quality ones.

### Relationship between SOM fractions and tobacco quality

The RDA and Monte Carlo permutation tests were performed to analyze the relationship between SOM compounds and the chemical composition of tobacco leaves (Fig. 7b,c). According to the standard, scores based on the tobacco quality evaluation level system were also included. Our results suggested that chemical components of tobacco leaves depended to a large degree on SOM composition.

For the RDA based on the chemical content of tobacco leaves (Fig. 7b), lipids, proteins, amino sugar, carbohydrates, and tannins had collective effects on tobacco quality. Together they explained 89.86% of the total variability. Amino sugar and tannins had a significant effect on the chemical components of tobacco leaves, explaining 59.2% and 22.5%, respectively. The content of N and nicotine in tobacco leaves were positively correlated to amino sugar while other contents were negative. The tannins were positively correlated with starch, nicotine content and nitrogen/nicotine ratio only. According to the position of different samples projected on the arrow ray of DOM composition, we could figure out their relationships. For the amino sugar, JS, DF, WN1, and NY were positively correlated to it while QX and WN2 had a negative relationship. The tannins positively impacted WN1, QX, DF, WN2 and negatively influenced JS, NY.

For the RDA analysis based on the score of each content in tobacco leaves (Fig.  c), carbohydrates, amino sugar, tannins, unsaturated hydrocarbons, and lipids differed in impact degrees and only carbohydrates had significant effects (*P* = 0.028). Both Axis 1 and 2 explained 97.36% of total variances. The scores of N, reducing sugar and nicotine content, nitrogen/nicotine and nicotine/reducing sugar ratio were positively related to carbohydrates while starch, K, and K/Cl had negative relationships. DF, WN1, NY, and QX were positively related to carbohydrates while JS, WN2 were opposite. Interestingly, the position of high-quality samples, WN1, NY and DF were closer than other samples, indicating their lower difference than low-quality ones.

## Discussion

Recently, FTICR-MS has been widely used to analyze SOM molecular composition. However, lots of researches focused on the molecular composition of humic, the change of SOM fraction after the fire or frozen^20,11^, and the comparison of organic matter fraction between stream and soil^21^. Our study was first to explore the relationship between SOM fraction and plant quality. The unbiased extraction from soils is difficult because the soil is one of the most complex and heterogeneous of all microbial environments. MeOH and CHCl_3_ can extract more fractions than water (Fig. 3), in accordance with other studies^22^. Besides, the groups of extracted fractions can be affected by the type of solvent as well. In our study, MeOH and CHCl_3_ can extract more lipids than water (Fig. 4), which may be due to organic solvents preferred compounds with long carbon chains such as lipids^23^. On the contrary, more lignin and condensed hydrocarbons were found in water extractions than in other fractions (Fig. 4). In addition, the O/C ratios of the water extractions were higher than those extracted by organic solvents (Table S2). The possible reasons are H_2_O has a very high polarity index and polar compounds, as well as compounds with high O/C ratios and oxygen number, are soluble in H_2_O^23^.

QX had the least formulas while WN1 and WN2 had the most, related to their meteorological conditions and geographical location (Table S1). The chemical and functional properties of SOM depend on its source material and biogeochemical history^24^. Many researchers also found that the diversity of SOM fraction is greatly affected by altitude, soil depth, vegetation, and microbial communities^25–29^.

According to the O/C and H/C ratio, SOM was mainly composed of lipids, unsaturated hydrocarbons, condensed hydrocarbons and lignin, consistent with other studies^12^. Differently, the SOM was mainly composed of lignin and condensed hydrocarbons in the Arctic soils but we detected more lipids and unsaturated hydrocarbons^11^. This is mainly because plants can change the microbial types and SOM compounds through may be affected by the plant growth and metabolism^30^. Thus, the SOM compounds based on physically defined fractions are increasingly used to interpret the dynamics of organic matter in the soil .

Moreover, by calculating the (H/C)w, (N/C)w, (O/C)w, C#w, (DBE)w, (Ai-mod)w, (DBE/O)w, (DBE/C)w, (DBE-O)w based on the formula of SOM fraction, we found that the (H/C)w, (N/C)w and (O/C)w of SOM were higher than that of DOM in rivers^31,32^. H/C indicates the degree of hydrogen saturation, the compounds extracted by non-polar solvents had a lower degree of hydrogen saturation than polar solvents. O/C describes the degree of oxygenation and the degree of nitrogen content is given by N/C. We can find out that hydrogen and oxygen saturation and nitrogen content were higher in soils than in rivers. Furthermore, the degree of oxygenation and nitrogen contents were higher in water extracts.

The chemical composition of tobacco leaves may be closely related to its quality while the top quality is gained when the chemical compositions are coordinated properly. In particular, the nicotine content, nitrogen content, and nitrogen/nicotine ratio are essential indicators of tobacco quality. The presence of nicotine significantly affects the taste of tobacco and may lead to the biting or light taste of tobacco if the nicotine content is not appropriate^33^. The optimum range of nicotine content in tobacco is about 2.2–2.8%. The nicotine content was highest in JS while lowest in WN2 and only WN1 complied with this standard. For the reason that these tobacco from six sites were of the same species (Yun 87) and growing in the same city, soil conditions may mainly affect their qualities. Due to the risk of nicotine on human health and the challenge of increasing regulation on the tobacco industry as well as its pollution to the atmosphere^34^, it is urgent to take measures to limit nicotine content to an appropriate range. Unlike nicotine, the N content is the most common deficiency in tobacco and can affect the yield and quality of tobacco leaves^35^. However, excessive nitrogen will reduce the content of phenolic substances and lignin in the plant and weaken the disease resistance of tobacco plants^36^. QX and WN2 had the least tobacco nitrogen content while their soils had the highest nitrogen content, indicating that only high nitrogen concentration in the soils did not directly lead to the high nitrogen concentration in tobacco plants. The nitrogen content in all sites did not exceed the standard for high-quality tobacco. Tobacco with a high smoke quality has an optimal nitrogen to nicotine ratio of 0.95–1.05, with 1 being the best. The nitrogen to nicotine ratio at all points exceeded the standard range and the main problem lied in the content of nicotine. The probable cause is that the soil total nitrogen was not suited for forming optimal nitrogen to nicotine ratios.

The carbohydrate content, such as sugar and starch is also crucial in evaluating the quality of tobacco and research found that consumers preferred high-level sugar tobaccos^37^ mainly because the sugar can improve the taste of tobacco^38^. The sugar and starch contents in JS, QX and WN2 were higher than the standard and led to their low quality (Fig. 2). Sugars in tobacco are formed by enzymatic hydrolysis of starch after initiation (harvesting) and early stages of the curing process^39^. The sugar content that is too low or too high can influence the taste, likely resulting in a more biting or acidic taste. The presence of starch in tobacco products can cause bitterness and flavor changes^40^. High quality tobacco has an optimal reducing sugar to nicotine ratio of 8.5–9.5, WN1, DF, and JS were lower than the ratio while NY, QX and WN2 were higher. The nitrogen content in soils from NY, QX and WN2 was higher than other three sites. We suggested that the nitrogen contents in these soils were too high for the tobacco.

A high potassium content in tobacco leaves can improve the combustibility, aroma, taste, and safety, and the potassium content plays a key role in the appearance and internal quality of tobacco leaves^41^. The K content and K/Cl ratio in our study were in the acceptable range.

For general crops, the higher the SOM content, the higher the soil fertility. But for tobacco, due to its special nitrogen requirement, too high or too low SOM content is not good for tobacco growth. When the SOM content is too high, the upper leaves cannot fall yellow normally, and even turn black, which is the appearance of the incoordination of chemical composition in the tobacco leaves. When the SOM content is too low, the tobacco plants grow short and leaves are small and thin. Only when the SOM content is appropriate, the tobacco leaves can have excellent appearance quality and internal quality. Many studies have documented that larger C/N ratios in soils indicate the presence of recent plant-derived particulate organic matter and a higher proportion of labile N in the soil^42^. Whereas smaller C/N ratios derive from microbial activities because they benefit from root exudates and the better living conditions evolved by the rhizosphere and enables them to colonize the deep mineral soil^43^. The C/N ratio in NY was significantly higher than other sites and DF was the lowest (Table 1). In our study, the SOM content in JS, QX and WN2 were significantly higher than other sites. However, the quality of tobacco was worse in JS, QX and WN2thanWN1, DF, and NY. Although their growing conditions were similar, the quality of tobacco of WN1 and WN2 showed significant differences (Fig. 2, Table S1). The N content, carbon content and SOM content in WN1 soil were all lower than those in WN2 soil, while the N content in the WN1 plant was higher than that in WN2 and the quality of WN1was better than WN2. According to the RDA results (Fig. 7a), WN1 and WN2 were separated by RDA1 and had a different relationship with SOM compounds. WN1 was positively related to amino sugar, proteins, lipids, tannins, carbohydrates, while WN2 had negative relation. Thus, we suggest that it is the SOM compounds but not the SOM and N content that influence the quality of tobacco primarily.

RDA results showed that amino sugar and tannins significantly influenced the chemical composition of tobacco leaves. Lipids, proteins, and amino sugars are relatively labile and known microbial metabolites and exudates that may be recycled and regenerated^30^. Those labile compounds can be easily employed by plants and are also the main factors affecting tobacco quality in our study. There were 105 same formulas among all the high-quality tobacco-growing soils and were mainly composed of lipids, unsaturated hydrocarbons and lignin (78.3%). These compounds may together affect the quality of tobacco but the regulatory mechanisms need further study.

## Conclusion

In this study, we firstly analyzed the relationship between tobacco quality and SOM molecular composition using FTICR-MS. SOM compounds were related to the chemical composition of tobacco leaves, which was consistent with our assumption. SOM compounds of high-quality tobacco were similar while were considerably different in low-quality tobacco. Each type of compound influenced the chemical composition of tobacco leaves differently. The amino sugar, proteins, lipids, tannins and carbohydrates together affected the chemical composition of tobacco leaves. Amino sugar and tannins, which were labile compounds, significantly affected the quality of tobacco. Thus, our study found the specific relationship between SOM compounds and tobacco leaves chemical composition, which can provide suggestions for improving tobacco quality sustainably.

## Materials and methods

### Study sites

Sampling sites were located in Bijie (103°36′–106°44′, 26°21′–27°47′), Guizhou Province, Southwest China (Fig. 1). The map was generated by the software Arcgis 10.3. The soil physical and chemical properties are listed in Table S1. Bijie covers an area of 26,848.51 km^2^ with a subtropical monsoon climate and a significant elevation difference in the territory, which is suitable for growing tobacco.

According to Bijie 2019 statistical yearbook, Bijie produces approximately 287,500 t of flue-cured tobacco on 3,730,000 ha of land every year. In terms of tobacco quality, the percentage of premium cigarettes is 72%, and the proportion of medium smoke is 28%. Based on the tobacco leaf quality in different areas of Bijie, six sampling points Wei Ning (WN1), Na Yong (NY), Da Fang (DF), Jin Sha (JS), Qian Xi (QX), and Wei Ning2 (WN2) were selected (Fig. 1, Table S1).

### Sample collection and preparation

At harvest, the topsoil samples (0–20 cm) and medium tobacco leaves were collected in July 2019. Soil samples were air-dried, ground, and then passed through a 2 mm sieve for laboratory analysis. The total carbon and nitrogen content were determined using an Elementar (Vario Micro, Elememtar Analysensysteme GmbH). The soil pH was measured in a water suspension at a soil–water ratio of 1:2.5 (w/v). SOM content was determined by the Walkley–Black wet digestion method^44^.

The fresh leaves were rinsed with deionized water and dried at 65℃ to constant weight for further analysis. The chemical composition of tobacco leaves, including nicotine, total nitrogen (N), potassium (K), chlorine (Cl), total sugar, reducing sugar, and starch were analyzed using continuous flow method. To evaluate the quality of tobacco, the evaluation system was used as follow. The comprehensive index (P) can be calculated by Eq. (1)^45^,

1 $$ P = \sum {C_{i} \cdot P_{i} } $$

P is the comprehensive index of the chemical composition of flue-cured tobacco; C_i_ is the quantitative score of the chemical component index; P_i_ is the relative weight of the chemical composition index.

### Solvent extraction for SOM

For FTICR-MS analysis, the sequential extraction protocol for SOM was followed the method of Tfaily^22^. Briefly, the bulk soil (100 mg) was put into a 2 mL glass centrifuge tube, adding 1 mL water and shaking for two hours on an Eppendorf Thermomixer before centrifugation at 12,000*g* for 10 min at room temperature. Then, we pulled off the supernatant and the soil residue was dried with nitrogen to remove any residual solvent. Then, 1 mL MeOH was added to the dried soil and repeat the above steps. CHCl_3_ solvent was added finally to complete the extraction.

### FTICR-MS data acquisition and data analysis

Molecular compositions of SOM in the soil extracts were determined by a 7.0 T FTICR-MS (Bruker Daltonics Inc., Billerica, MA, USA) fitted with a standard electrospray ionization (ESI) interface. The instrument was externally calibrated weekly to a mass accuracy of < 0.1 ppm using a tuning solution from Agilent. The CHCl3 and H2O extracts were diluted in MeOH to improve ESI efficiency and was injected to the ESI source with 240 μL/h and the positive ESI needle voltage was set to − 4500 V. Blanks (HPLC grade MeOH) were also analyzed at the beginning and the end of the day to monitor potential carry over from one sample to another. The instrument was flushed between samples using a mixture of water and methanol.

Chemical formulas were assigned based on the following criteria: S/N > 7, and mass measurement error ≤ 1 ppm, taking into consideration the presence of C, H, O, N, K, and Na and excluding other elements (C_1-100_, H_1-200_, N_0-7_, O_0-50_, H/C ≤ 2.5, N/C ≤ 0.5, O/C ≤ 1.5)^46^. Additionally, several peaks with multiple possible candidate formulas were assigned formulas with the lowest error with the lowest number of heteroatoms.

For statistical analyses, we calculated a set of magnitude-weighted parameters based on each formula and relative intensity, including oxygen to carbon ratio (O/C), hydrogen to carbon ratio (H/C), nitrogen to carbon ratio (N/C), aromaticity index (AI-mod), double bond equivalents (DBE)^8^, double bond equivalents to carbon ratio (DBE/C), double bond equivalents to oxygen ratio (DBE/O), the difference between DBE and number of oxygen (DBE-O) and number of carbon atoms (C#) to characterize each sample. In addition, we calculated the AI-mod and DBE from the number of atoms according to Eqs. (2) and (3).

2 $$ {\text{DBE}} = {1} + \left( {{\text{2C}}{-}{\text{H}} + {\text{N}}} \right)/{2} $$

3 $$ {\text{AI}} = \left( {{1} + {\text{C}} - {\text{O}}/{2} - {\text{H}}/{2}} \right)/\left( {{\text{C}} - {\text{O}}/{2} - {\text{N}}} \right) $$

The magnitude-weighted parameters were calculated for each measurement as the sum of all of the products of chemical information and relative intensity (e.g., (C#)w = ∑(C#n ∗ Mn) with n for each elemental formula and M for the relative peak intensity^21,31^.

DBE represents the degree of unsaturation by providing the number of $\uppi$ bonds and rings in a molecule. High DBE/C ratios can represent a high density of C=C double bonds and reveal aromatic or condensed aromatic structures^8^. Moreover, DBE-O is used to describe C=C unsaturation by excluding all the possible C=O bonds that are abundant in RCOOH functionalities^47^. AI-mod is applied to distinguish between non-aromatic (AI-mod ≤ 0.5), aromatic (AI-mod > 0.5) and condensed aromatic (AI-mod ≥ 0.67) compounds. DBE/O is inversely related to the possible number of C/O bonds. Besides, H/C indicates the degree of hydrogen saturation, the degree of oxygenation is described by O/C, and N/C gives the degree of nitrogen content.

In this study, the chemical compounds were grouped into the eight main families: lipids (0 < O/C ≤ 0.3, 1.5 ≤ H/C ≤ 2.5), proteins (0.3 < O/C ≤ 0.55, 1.5 ≤ H/C ≤ 2.3), amino sugars (0.55 < O/C ≤ 0.7, 1.5 ≤ H/C ≤ 2.2), carbohydrates (0.7 < O/C ≤ 1.5, 1.5 ≤ H/C ≤ 2.5), unsaturated hydrocarbons (0 ≤ O/C ≤ 0.125, 0.8 ≤ H/C ≤ 1.5), lignin (0.125 < O/C ≤ 0.65, 0.8 ≤ H/C < 1.5), tannins (0.65 < O/C ≤ 1.1, 0.8 ≤ H/C < 1.5), and condensed hydrocarbons (0 ≤ O/C ≤ 0.95, 0.2 ≤ H/C ≤ 0.8)^12^.

### Statistical analysis

A one-way analysis of variance (ANOVA) followed by Waller-Duncan test was applied to determine the significant differences (*P* < 0.05) of SOM content, nitrogen, carbon content in the soils, and chemical composition in tobacco leaves among different samples. Pearson correlation coefficients were used to describe the relationships between the chemical composition of the tobacco leaves and soil factors. We used principal component analysis (PCA) and redundancy analysis (RDA) with forwarding selection to compare our data and determine the relationship between tobacco chemical composition and SOM compounds classification.

### Statement

The collection of the tobacco leaves was under the permission from the local suppliers. The authors confirm that all methods were performed in accordance with the relevant guidelines and regulations.

## Supplementary Information

Supplementary Information.

## Data Availability

All data generated or analyzed during this study are included in this published article (and its Supplementary Information files).
