# Supplementary material for: Molecular composition of soil organic matter (SOM) regulate qualities of tobacco leaves
Source: Sci Rep. 2022 Sep 12;12:15317. doi: 10.1038/s41598-022-19428-6 (PMC9468172; doi:10.1038/s41598-022-19428-6)
Supplement: Supplementary file 1 — Supplementary Information. [file 41598_2022_19428_MOESM1_ESM.docx]

***Supporting Information:***

**Molecular composition of soil organic matter (SOM) regulate qualities of tobacco leaves**

**Xu Zhai**^a#^**, Long Zhang**^b#^**, RuofanWu**^a^**, Mei Wang** ^a^**, Yanxiang Liu** ^b^**, Jiapan Lian**^a^**, Mehr Ahmed Mujtaba Munir** ^a^**, Dan Chen** ^a^**, Lei Liu** ^a^**, Xiaoe Yang** ^a*^

a. Key Laboratory of Environment Remediation and Ecological Health, Ministry of Education, College of Environmental Resource Sciences, Zhejiang University, 310058 Hangzhou, China；

b. Bijie Branch Company of Guizhou Tobacco Company, Guizhou 551713, China;

Xu Zhai and Long Zhang contributed equally to this work.

***Corresponding author:** Xiaoe Yang

**Full postal Address:** College of Environmental Resource Sciences, Zhejiang University, 310058 Hangzhou, China

**E-mail:** xeyang@zju.edu.cn

**Table S1.**Sampling sites information and soil physical and chemical properties.

| Sample | Location | Altitude(m) | Terrain | Soil Color | pH | CEC | Annual rainfall/mm | Annual sunshine/h | Annual average temperature (°C) |
| --- | --- | --- | --- | --- | --- | --- | --- | --- | --- |
| WN1 | Wei Ning | 2071.33 | flat | Yellow-Brown | 5.22c | 4.24b | 835.30 | 1577.70 | 11.40 |
| NY | Na Yong | 1532.78 | mountain | Yellow | 4.55d | 13.79a | 1321.90 | 1182.20 | 14.20 |
| DF | Da Fang | 1465.19 | mountain | Yellow | 4.35d | 1.80c | 1065.80 | 1296.00 | 12.50 |
| JS | Jin Sha | 934.99 | flat | Yellow | 5.82b | 5.85b | 1003.60 | 1032.20 | 15.80 |
| QX | Qian Xi | 1218.36 | flat | Yellow | 7.00a | 13.08a | 948.70 | 1128.60 | 14.90 |
| WN2 | Wei Ning | 2095.48 | mountain | Red | 5.21c | 5.67b | 835.30 | 1577.70 | 11.40 |

**Table S2. Magnitude-weighted parameters.**

| Sample |  | Extracts | (H/C)w | (N/C)w | (O/C)w | C#w | (DBE)w | (Ai-mod)w | (DBE/O)w | (DBE/C)w | (DBE-O)w | non-aromatic (%) | aromatic (%) | condensed aromatic (%) | |
| --- | --- | --- | --- | --- | --- | --- | --- | --- | --- | --- | --- | --- | --- | --- | --- |
| WN1 |  | H_2_O | 1.16 | 0.12 | 0.31 | 33.25 | 16.53 | 0.48 | 3.63 | 0.52 | 7.96 | 60.25 | 15.53 | 24.22 |  |
|  |  | MeOH | 1.36 | 0.09 | 0.28 | 41.34 | 16.20 | 0.34 | 4.34 | 0.39 | 6.02 | 78.25 | 6.86 | 14.89 |  |
|  |  | CHCl_3_ | 1.42 | 0.05 | 0.19 | 45.93 | 16.12 | 0.28 | 3.99 | 0.34 | 8.53 | 84.66 | 8.52 | 6.82 |  |
|  |  | Average | 1.37 | 0.07 | 0.24 | 42.82 | 16.19 | 0.32 | 4.11 | 0.38 | 7.37 | 75.02 | 9.94 | 15.04 |  |
| NY |  | H_2_O | 0.99 | 0.11 | 0.28 | 31.74 | 18.76 | 0.57 | 5.31 | 0.60 | 10.83 | 48.87 | 13.57 | 37.56 |  |
|  |  | MeOH | 1.18 | 0.07 | 0.17 | 43.30 | 20.86 | 0.42 | 4.68 | 0.47 | 13.75 | 86.86 | 4.49 | 8.65 |  |
|  |  | CHCl_3_ | 1.44 | 0.08 | 0.24 | 43.54 | 16.18 | 0.27 | 3.23 | 0.35 | 6.56 | 78.05 | 10.57 | 11.38 |  |
|  |  | Average | 1.30 | 0.08 | 0.22 | 42.54 | 18.22 | 0.35 | 3.96 | 0.42 | 9.70 | 73.30 | 8.99 | 17.72 |  |
| DF |  | H_2_O | 1.11 | 0.12 | 0.30 | 32.03 | 16.72 | 0.49 | 4.59 | 0.54 | 8.76 | 55.56 | 12.12 | 32.32 |  |
|  |  | MeOH | 1.39 | 0.09 | 0.23 | 41.46 | 16.28 | 0.33 | 4.33 | 0.38 | 8.13 | 78.54 | 9.01 | 12.45 |  |
|  |  | CHCl_3_ | 1.46 | 0.05 | 0.18 | 46.76 | 15.24 | 0.26 | 3.77 | 0.32 | 8.11 | 81.89 | 9.06 | 9.06 |  |
|  |  | Average | 1.40 | 0.07 | 0.21 | 43.31 | 15.81 | 0.31 | 4.08 | 0.36 | 8.17 | 71.15 | 10.13 | 18.73 |  |
| JS |  | H_2_O | 1.65 | 0.18 | 0.20 | 34.53 | 10.83 | 0.22 | 3.08 | 0.30 | 4.80 | 73.35 | 9.90 | 16.75 |  |
|  |  | MeOH | 1.68 | 0.09 | 0.11 | 43.18 | 10.39 | 0.18 | 3.43 | 0.23 | 6.27 | 83.58 | 5.57 | 10.85 |  |
|  |  | CHCl_3_ | 1.67 | 0.14 | 0.23 | 36.91 | 10.61 | 0.20 | 2.93 | 0.27 | 2.95 | 77.31 | 10.82 | 11.87 |  |
|  |  | Average | 1.67 | 0.12 | 0.18 | 39.33 | 10.53 | 0.19 | 3.15 | 0.25 | 4.50 | 77.83 | 8.89 | 13.29 |  |
| QX |  | H2O | 1.26 | 0.11 | 0.26 | 33.15 | 15.69 | 0.42 | 3.64 | 0.46 | 8.29 | 54.09 | 18.24 | 27.67 |  |
|  |  | MeOH | 1.22 | 0.08 | 0.19 | 45.43 | 20.82 | 0.42 | 3.85 | 0.46 | 14.03 | 69.81 | 7.55 | 22.64 |  |
|  |  | CHCl3 | 1.43 | 0.06 | 0.18 | 47.02 | 16.47 | 0.29 | 5.44 | 0.34 | 8.90 | 82.86 | 10.48 | 6.67 |  |
|  |  | Average | 1.34 | 0.07 | 0.19 | 45.32 | 18.00 | 0.35 | 4.71 | 0.39 | 10.73 | 67.14 | 12.29 | 20.57 |  |
| WN2 |  | H_2_O | 0.93 | 0.13 | 0.40 | 27.51 | 16.70 | 0.73 | 4.02 | 0.65 | 8.27 | 45.30 | 9.76 | 44.95 |  |
|  |  | MeOH | 1.34 | 0.08 | 0.18 | 43.06 | 17.54 | 0.33 | 5.76 | 0.40 | 11.09 | 82.32 | 6.35 | 11.33 |  |
|  |  | CHCl_3_ | 1.41 | 0.07 | 0.19 | 44.66 | 16.47 | 0.31 | 3.61 | 0.35 | 8.64 | 80.94 | 8.29 | 10.77 |  |
|  |  | Average | 1.35 | 0.08 | 0.20 | 42.87 | 16.89 | 0.35 | 4.45 | 0.39 | 9.54 | 71.32 | 8.01 | 20.67 |  |

The letter ‘w’ represents the magnitude-weighted parameters.

**
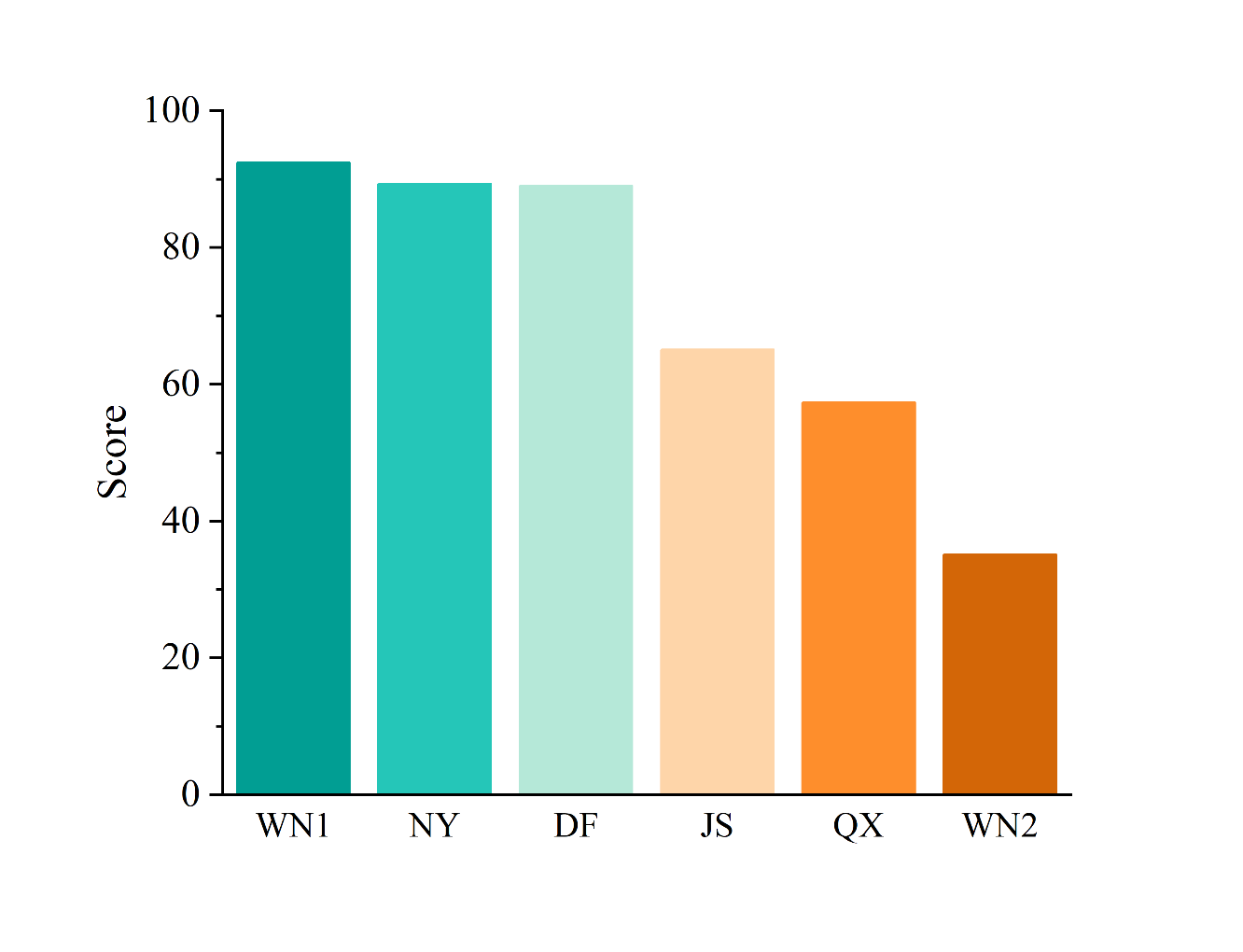
**

**Figure S1.The score of six tobacco quality calculated by the evaluation system.**
